# DNA-methylation-based telomere length estimator: comparisons with measurements from flow FISH and qPCR

**DOI:** 10.18632/aging.203126

**Published:** 2021-06-03

**Authors:** Emily E. Pearce, Steve Horvath, Shilpa Katta, Casey Dagnall, Geraldine Aubert, Belynda D. Hicks, Stephen R. Spellman, Hormuzd Katki, Sharon A. Savage, Rotana Alsaggaf, Shahinaz M. Gadalla

**Affiliations:** 1 Clinical Genetics Branch, Division of Cancer Epidemiology and Genetics, National Cancer Institute, National Institutes of Health, Rockville, MD 20850, USA; 2 Department of Biostatistics, Fielding School of Public Health, University of California School of Public Health, Los Angeles, CA 90095, USA; 3 Department of Human Genetics, David Geffen School of Medicine, University of California, Los Angeles, CA 90095, USA; 4 Cancer Genomics Research Laboratory, Division of Cancer Epidemiology and Genetics, National Cancer Institute, National Institutes of Health, Rockville, MD 20850, USA; 5 Leidos Biomedical Research, Inc., Frederick National Laboratory for Cancer Research, Frederick, MD 21701, USA; 6 Terry Fox Laboratory, British Columbia Cancer Agency, Vancouver, BC V5Z 1L3, Canada; 7 Repeat Diagnostics Inc, North Vancouver, BC V7M 1A5, Canada; 8 Center for International Blood and Marrow Transplant Research, Medical College of Wisconsin, Milwaukee, WI 53226, USA; 9 Biostatistics Branch, Division of Cancer Epidemiology and Genetics, National Cancer Institute, National Institutes of Health, Bethesda, MD 20892, USA

**Keywords:** telomere length, qPCR, flow FISH, DNAmTL, agreement

## Abstract

Telomere length (TL) is a marker of biological aging associated with several health outcomes. High throughput reproducible TL measurements are needed for large epidemiological studies. We compared the novel DNA methylation-based estimator (DNAmTL) with the high-throughput quantitative PCR (qPCR) and the highly accurate flow cytometry with fluorescent *in situ* hybridization (flow FISH) methods using blood samples from healthy adults. We used Pearson’s correlation coefficient, Bland Altman plots and linear regression models for statistical analysis. Shorter DNAmTL was associated with older age, male sex, white race, and cytomegalovirus seropositivity (p<0.01 for all). DNAmTL was moderately correlated with qPCR TL (N=635, r=0.41, *p* < 0.0001) and flow FISH total lymphocyte TL (N=144, r=0.56, *p* < 0.0001). The agreements between flow FISH TL and DNAmTL or qPCR were acceptable but with wide limits of agreement. DNAmTL correctly classified >70% of TL categorized above or below the median, but the accuracy dropped with increasing TL categories. The ability of DNAmTL to detect associations with age and other TL-related factors in the absence of strong correlation with measured TL may indicate its capture of aspects of telomere maintenance mechanisms and not necessarily TL. The inaccuracy of DNAmTL prediction should be considered during data interpretation and across-study comparisons.

## INTRODUCTION

Telomeres consist of tandem DNA nucleotide repeats (TTAGGG)n and a protein complex that cap chromosome ends to ensure chromosomal stability [1]. Telomeres shorten as cells divide, eventually leading to replicative senescence and/or apoptosis, making telomere length (TL) a useful marker of cellular and thus biological age [2, 3]. TL has been associated with a variety of age-related diseases and health outcomes including cardiovascular disease, metabolic syndrome, and cancer (reviewed in [4]). TL is used clinically to diagnose patients with inherited telomere biology disorders such as dyskeratosis congenita and has shown promise in guiding donor selection for hematopoietic cell transplant (HCT) [5–8].

Several methods have been developed for measuring TL, each with its own strengths and limitations [9, 10]. The current gold standard is the Southern blot Telomere Restriction Fragment (TRF) method; it measures average absolute TL (in kilobases, kb), and requires large quantities of high-quality DNA [11]. Another accurate method is fluorescence *in situ* hybridization (flow FISH) in which fluorescently labeled peptide nucleic acid (PNA) probes detect telomeric repeats in total leukocytes and leukocyte subsets to determine average TL as calibrated using TRF and presented in kb. This method requires viable leukocytes and special expertise [10, 12]. A widely used method is quantitative polymerase chain reaction (qPCR) that measures TL based on the ratio between telomere copy number and that of a single-copy gene (T/S) in the same DNA sample [13, 14]. qPCR is frequently used to determine TL in epidemiologic studies because of its high-throughput and small DNA requirements; however, its reliability is limited by its high sensitivity to pre-analytic factors, such as DNA extraction or storage [15]. Other methods target the shortest telomeres such as single telomere length analysis (STELA) and the Telomere Shortest Length Assay (TeSLA) [16, 17]. Large scale genomic and epigenomic data offer opportunities for new approaches to TL calculation, such as TelSeq, an open-source software that is correlated with Southern blot (r~0.6) and estimates TL in kb using whole-genome sequence data [18].

Existing methods and high throughput adaptations have extended telomere research to population-level studies; however, there remains a need for TL measurement tools that overcome the limitations of current techniques: the sample quantities and analysis time required by the most accurate methods, and the limited reliability of high-throughput methods. DNA methylation regulates gene expression and has been associated with both chronological age and telomere shortening [19–21]. A new method utilizing whole genome DNA methylation array data to predict TL in kb was recently introduced [22] and may be useful to explore TL questions using available methylation array databases. This study aims to independently evaluate the performance of DNAmTL in comparison with TL measured by flow FISH and qPCR and evaluate the relationship between DNAmTL and participant characteristics known to be associated with TL.

## RESULTS

### Participant characteristics

The study included 635 healthy adults (median age=34 years, range=19-61) who were HCT donors; blood samples were available at the Center for International Blood and Marrow Transplant Research (CIBMTR) biorepository and were part of the National Cancer Institute (NCI) Transplant Outcomes in Aplastic Anemia (TOAA) Study. Of the 635 individuals, 425 (67%) were male, 478 (75%) were white, and 239 (38%) were cytomegalovirus (CMV) seropositive (Table 1).

**Table 1:** Characteristics of study participants.

**Variable**	**Total (n=635) N (%)**
Age	
19-24	118 (19%)
25-29	124 (19%)
30-34	96 (15%)
35-39	107 (17%)
40-44	91 (14%)
45-61	99 (16%)
Sex	
Male	425 (67%)
Female	210 (33%)
Race	
White	478 (75%)
African American	40 (6%)
Other	93 (15%)
Missing	24 (4%)
CMV serostatus	
Positive	239 (38%)
Negative	370 (58%)
Missing	26 (4%)
**Telomere length (TL)**	**Median (Range)**
DNAmTL (kb)	7.4 (6.6-8.4)
flow FISH TL (kb)^1^	7.0 (3.7-11.2)
qPCR (z-score)	-0.17 (-2.34-4.96)

^1^flow FISH TL was available for 144 participants. Abbreviations: CMV indicates cytomegalovirus; DNAmTL (kb) indicates DNA methylation-based estimator of telomere length in kilobases; flow FISH TL (kb) indicates lymphocyte telomere length measured by fluorescent *in situ* hybridization with flow cytometry in kilobases; qPCR (z-score) indicates calculated z-score of telomere length measured by quantitative polymerase chain reaction.

### Association between DNAmTL and TL-related participant characteristics

**Figure 1:**
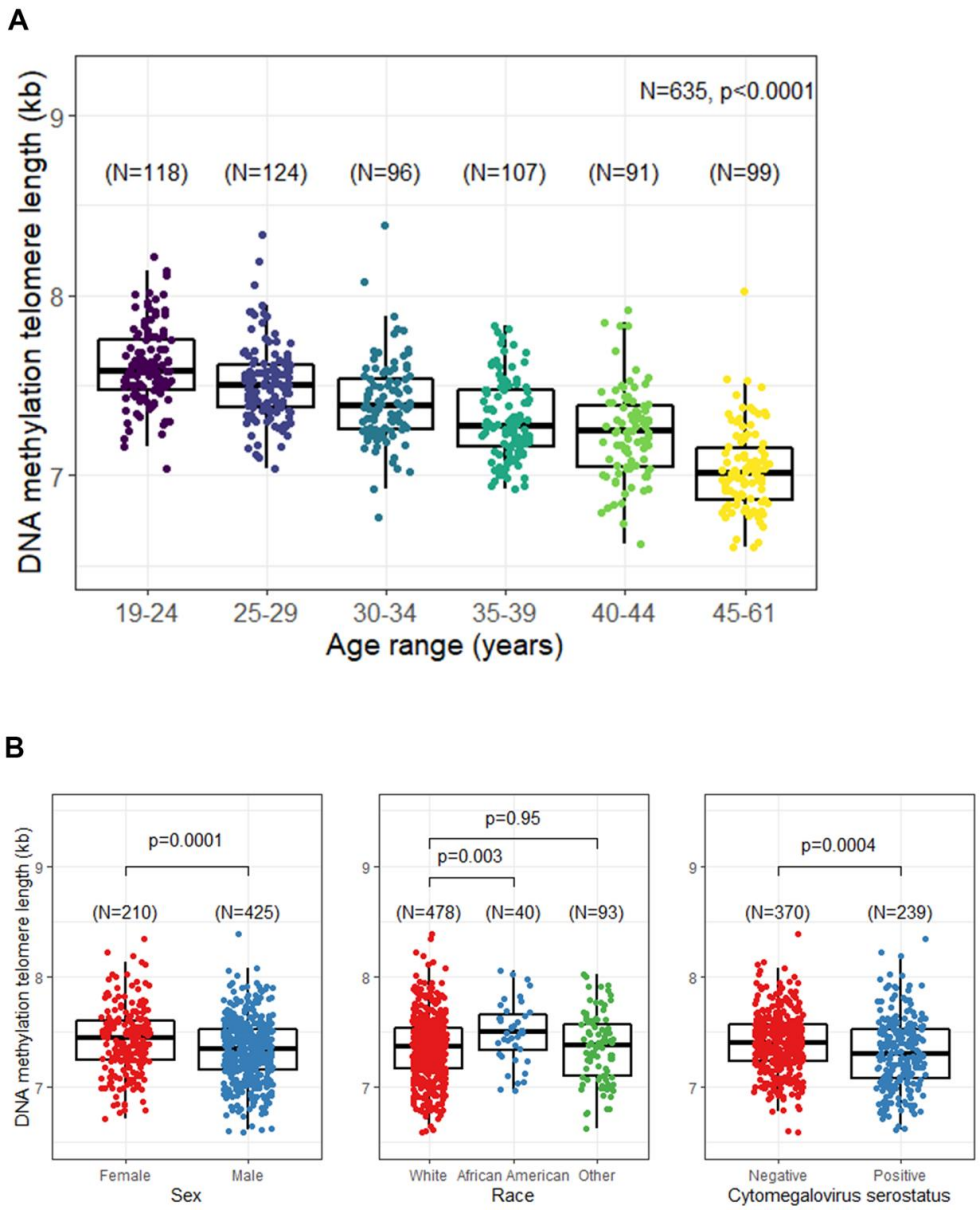
**Unadjusted relationship between DNA methylation telomere length (DNAmTL) and selected participant characteristics.** (**A**) DNAmTL by age categories. (**B**) DNAmTL by sex, race, or cytomegalovirus (CMV) serostatus.

A statistically significant strong negative association between DNAmTL and chronological age was noted; the correlation coefficient (r) = -0.65, p<0.0001(Figure 1A). Shorter DNAmTL was associated with male sex (p=0.0001) and CMV positive serostatus (p=0.0004). African Americans had longer DNAmTL compared with other race groups; the difference between TL in African Americans and Whites was statistically significant (p=0.003), but no difference was noted between Whites and other race groups (p=0.95) (Figure 1B). In a multivariable regression model including all previously tested variables, DNAmTL decreased by 21 bp per year (p<0.0001), and was 116 bp longer in women than men (p<0.0001), 213 bp longer in African Americans than whites (p<0.0001), and 83 bp shorter in CMV seropositive individuals than those who were seronegative (p<0.0001) (Table 2).

**Table 2:** Adjusted associations between DNAmTL and selected participant characteristics.

**Variables^1^**	**β**	**p-value**
**Age**	-0.021	<0.0001
**Sex**		
Male	REF	
Female	0.116	<0.0001
**Race**		
White	REF	
African American	0.213	<0.0001
Other	0.010	0.68
**CMV serostatus**		
Negative	REF	
Positive	-0.083	<0.0001

^1^All variables in the table were included in the same model.

Abbreviation: CMV indicates cytomegalovirus.

### DNAmTL reliability and comparisons with qPCR or flow FISH TL

In 48 samples with blinded duplicate of MethylationEpic array data, the mean coefficient of variation (CV) of calculated DNAmTL was 1% (range= 0.08-3.1%). These blinded duplicate samples also had high DNAmTL correlation (r=0.93, p<0.0001).

**Figure 2:**
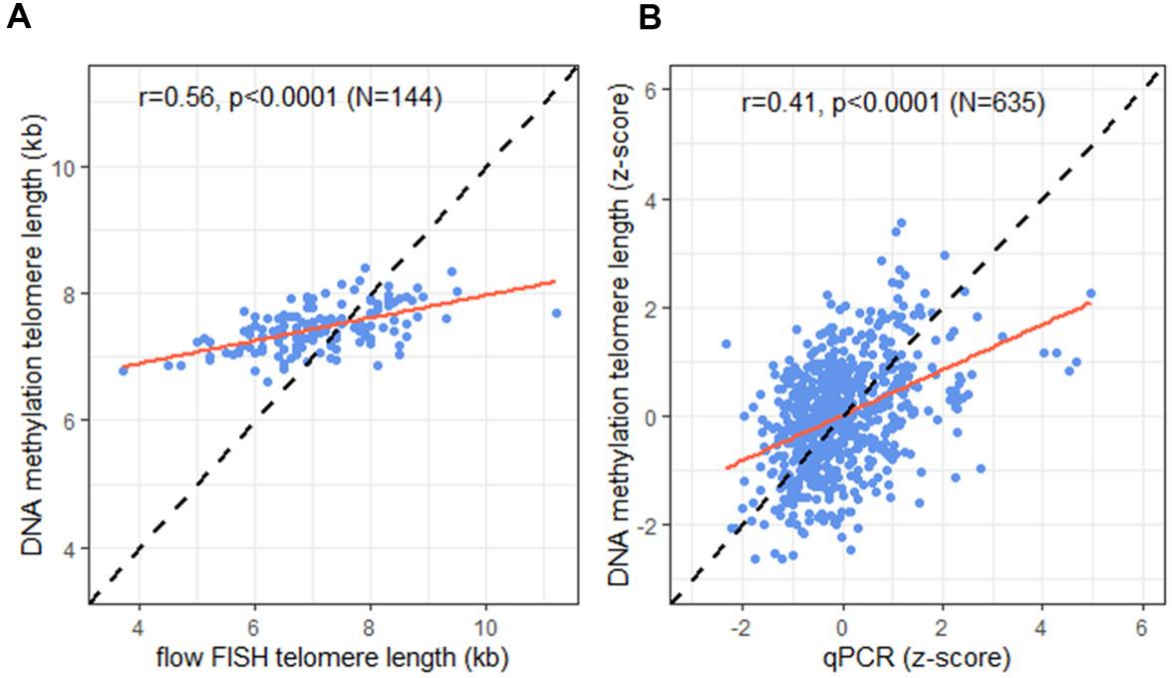
**Correlation of telomere length (TL) measurement tools.** (**A**) DNA methylation telomere length (DNAmTL) and flow FISH TL in kilobases (kb); (**B**) DNAmTL and qPCR in z-scores. Dashed line represents perfect agreement. Solid red line represents regression line.

Comparison of the TL estimated by DNAmTL and measured by flow FISH in the 144 individuals with both measures showed a statistically significant difference (median 7.4 *vs.* 7 kb respectively, p<0.0001), and moderate correlation (r = 0.56, *p* <0.0001; Figure 2A). In the full cohort (N=635), predicted TL by DNAmTL and qPCR measured TL were also statistically significantly different (median z-score TL =0.05 *vs.* -0.17 standard deviations from the mean, respectively, p=0.03), and showed modest correlation (r=0.41, p<0.0001; Figure 2B). No statistically significant differences in the correlations between TL from DNAmTL and qPCR were noted by sex (p-interaction=0.31), race (p-interaction=0.10), or age (p-interaction=0.11).

**Figure 3:**
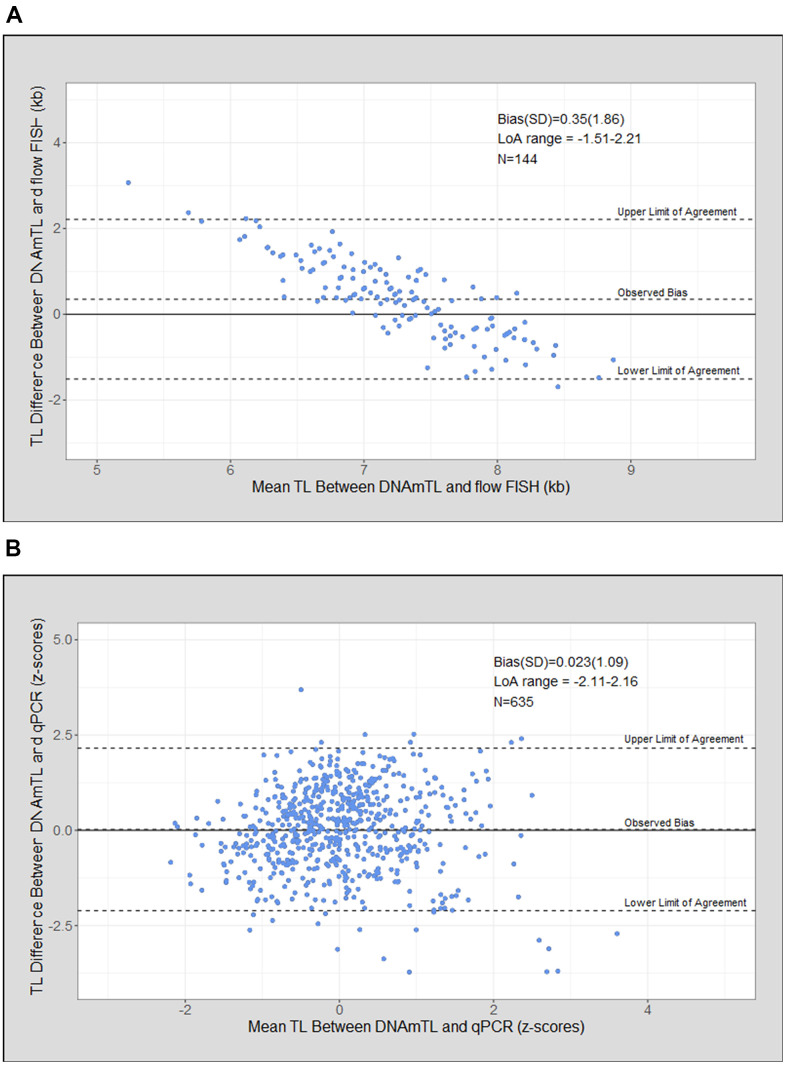
**Telomere length (TL) measurement agreement between measurment tools.** (**A**) DNA methylation telomere length (DNAmTL) and flow FISH in kilobases (kb); (**B**) DNAmTL and qPCR in z-scores.

Bland Altman analysis of DNAmTL compared with flow FISH TL demonstrated a mean bias of 0.35 kb (standard deviation [SD]=1.86), and a wide limit of agreement (LoA = -1.51 to 2.21 kb). DNAmTL resulted in a narrower range of TL compared with flow FISH with overestimation of the shortest TL and underestimation of the longest (Figure 3A). The mean bias was 0.023 (SD= 1.09) for DNAmTL and qPCR TL (Figure 3B).

**Figure 4:**
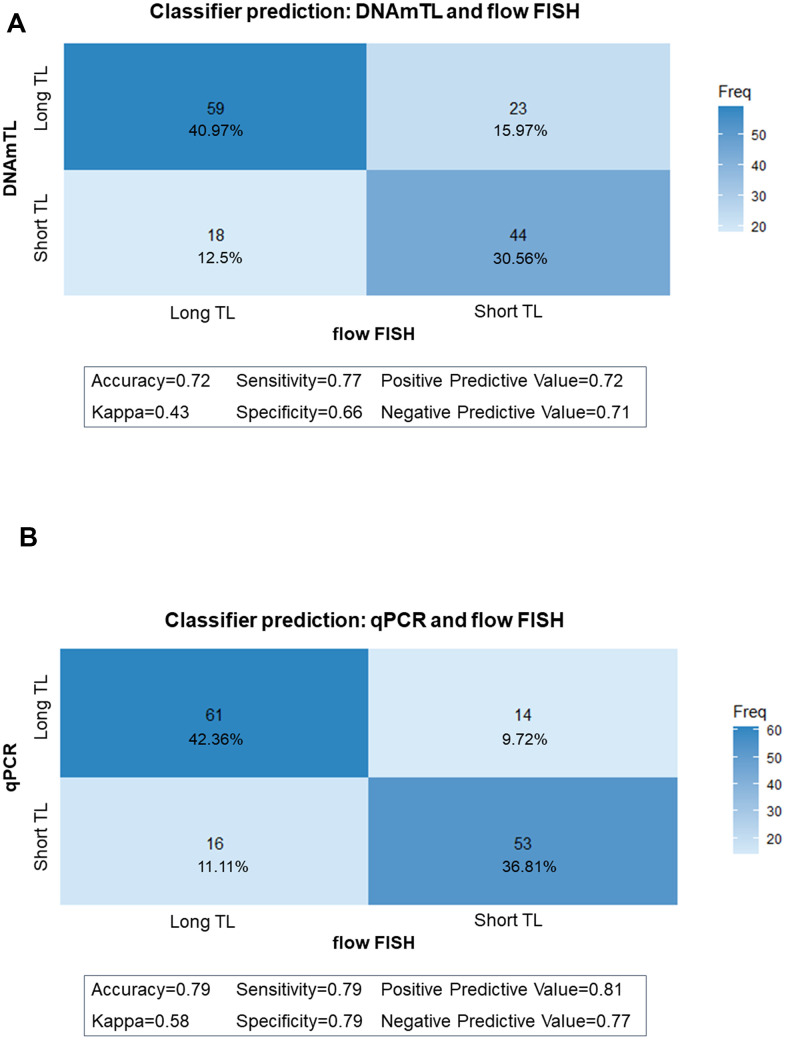
**Classifier matrices comparing telomere length (TL) categories (above and below median).** (**A**) between flow FISH and DNA methylation telomere length (DNAmTL); (**B**) between flow FISH and qPCR.

We then used classifier matrices to assess the agreement between DNAmTL or qPCR TL with that of flow FISH when categorized as long *versus* short based on the TL median. DNAmTL correctly classified 72% of the individuals as having long (TL above median) or short (TL below the median) with 77% sensitivity and 66% specificity, relative to flow FISH. Similarly, agreement between qPCR and flow FISH showed 79% accuracy, 79% sensitivity, and 79% specificity (Figure 4A, 4B). When the analysis was repeated to evaluate the ability of DNAmTL or qPCR to correctly classify TL in tertiles or quartiles, accuracy of both methods decreased. For DNAmTL, the TL accuracy for the tertile classifier=57%, and for the quartile classifier =42%. Similar results were noted with qPCR TL (tertile accuracy=67%, quartile accuracy=52%). Of note, both DNAmTL and qPCR TL showed highest agreement with flow FISH at the longest and shortest TL categories for both tertiles and quartiles.

## DISCUSSION

In this study, we found that DNAmTL detected the expected variations in TL by age, sex, race, and CMV serostatus (a marker of chronic infection). However, its correlation with TL measured by flow FISH or qPCR was modest and the limits of measurement agreement were wide. On the binary scale, both DNAmTL and qPCR correctly classified approximately two-thirds of the individuals into long or short TL (cutoff at the median) when tested against flow FISH. The accuracy of both methods declined when TL was classified into more than two categories. These results suggest there may be opportunities for using methylation array data to explore TL variability in large epidemiological studies but call for caution in directly comparing DNAmTL results with standard TL measurement methods because of its limited accuracy.

The current study showed a modest correlation between DNAmTL and flow FISH (r=0.56), or qPCR (r=0.41). A previously published report showed a consistent correlation between DNAmTL and TRF (N of datasets=4, N of samples = 4788, range of r= 0.41- 0.5), but the correlation between DNAmTL and qPCR was variable (N of datasets=3, N of samples = 2136, range of r= -0.01- 0.38) [22]. This may reflect the known lab-to-lab variation for qPCR TL assay or be affected by assay reproducibility. Here, we showed a mean CV for DNAmTL of 1% indicating a high reproducibility of TL estimates based on 48 replicates. A previous study showed a CV of 1.7% for TRF and >5% for qPCR [23].

In line with previous studies using the Bland Altman analysis comparing agreements between TL measurement methods [24–26], DNAmTL showed acceptable agreement with TL measured by qPCR (mean bias ± SD = 0.023 ± 1.09) or flow FISH assay (mean bias ± SD = 0.35 ± 1.86) with almost all observations falling within two standard deviations for the limits of agreement. However, the observed wide limits of agreement (*e.g.,* -1.51 to 2.21 kb in the flow FISH comparison) reflect a lack of accuracy that may limit the applicability of DNAmTL. When evaluating the ability of DNAmTL or qPCR to accurately categorize TL as compared with flow FISH, the study showed attenuated accuracy as the number of categories increased. This may highlight the importance of taking assay measurement error into account when calculating the minimum required sample size for new studies to be able to detect significant differences [27, 28].

Despite the modest correlation between DNAmTL and measured TL by evaluated assays, DNAmTL showed the expected negative TL-age correlation. Of note, the observed DNAmTL correlation with age was stronger (r= -0.64) than that reported with other TL measurement assays in this dataset (r = -0.30 for qPCR TL, and r= -0.33 for flow FISH TL) [25]. Additionally, in a meta-analysis of 124 cross-sectional and 5 longitudinal studies, the pooled TL-age correlation for TRF was r= -0.34 and for qPCR r= -0.29 [29]. On the other hand, comparisons of DNAmTL differences across age groups in the current cross-sectional study suggested that for every year increase in age, DNAmTL decreased by 21 bp. This is slightly lower than other methods in which a decrease of 30-60 bp per year was detected [30]. Notably, the majority of previous literature on TL dynamics also used a cross-sectional approach; longitudinal studies with serial samples are needed to address this question. DNAmTL also detected known TL relationships with sex, race [31, 32] and chronic infections [33, 34]. Of interest, DNAmTL in the current study detected greater TL differences by race and sex than those reported by TRF in a study including 1510 individuals (of whom 142 were African Americans and 888 were females) [35]. This may be influenced by sex- or race-specific methylation differences [36]. Yet, it is also possible that the relatively small sample size of certain subgroups in our study may have resulted in imprecise estimates. DNAmTL also demonstrated the expected associations with BMI and smoking behavior in another study [22]. These observations, despite modest correlations with other methods, support that DNAmTL may be capturing a broader aspect of the biological processes underlying cellular aging than just telomere shortening. Previously reported *in vitro* examination of cultured somatic cells showed that DNAmTL captured cellular proliferation independent of telomere attrition and telomerase activity [22]. Therefore, more robust associations between DNAmTL and TL-related factors that may themselves be more strongly linked to cellular aging than to telomere shortening are biologically plausible and point to the utility of future studies of DNAmTL in capturing such variations.

The strengths of the current study included the availability of TL measurements from multiple assays allowing for a comprehensive comparison with the new DNAmTL estimator in a relatively large sample size of healthy individuals. Flow FISH data provided an accurate TL measurement comparison with DNAmTL, and qPCR data allowed us to compare DNAmTL to a method widely used in epidemiological studies. Our study was limited by the age range (19-61 years), so our estimates may not be generalizable to all ages. Additionally, our DNAmTL associations with TL-known factors were not adjusted for other possible confounders such as socioeconomic status, smoking, or BMI, due to lack of information. However, the association between these factors and TL is not firmly established [37–40]. In addition, the study population consisted of pre-screened, healthy, HCT donors, therefore these factors are unlikely to have significant effects on our results. Although we measured TL using several assays (flow FISH and qPCR), we were limited by the absence of TL measurement by TRF, the gold standard in TL research. Of note, a strong correlation between TL measured by flow FISH and TRF has been reported (R^2^ = 0.73; p < 0.001) [41].

In conclusion, the results of this study suggest that DNAmTL holds promise as a method for approximating TL as long as its limitations are understood. In the current era of genomic data sharing there is an opportunity to use DNAmTL to explore different avenues of telomere and aging research using methylation data in the public domain. The sensitivity of DNAmTL in detecting known TL associations and its ability to approximate average TL suggest it may have utility in epidemiologic studies. However, because DNAmTL correlation and agreement with other methods is only modest, researchers should exercise caution when using DNAmTL in contexts where accuracy is of primary importance. Additional studies are needed to better understand what specific aspects of cellular aging or telomere maintenance are being captured by DNAmTL.

## MATERIALS AND METHODS

### Study participants

The study participants were HCT donors in the TOAA project, a collaboration between NCI and CIBMTR [42]. Blood samples (frozen whole blood (N=416), or peripheral blood mononuclear cells (PBMC; N=219) collected before hematopoietic stem cell donation were available at the CIBMTR biorepository (https://www.cibmtr.org/Pages/index.aspx).

### Telomere length measurement

For the flow FISH assay, total leukocytes were isolated from cryopreserved PBMCs. The fluorescence intensity in total lymphocytes and lymphocyte subsets, defined by labeled antibodies specific for CD20, CD45RA and CD57, relative to internal control cells and unstained controls were measured on a FACSCalibur instrument (Becton Dickinson) to calculate the median telomere length from duplicate measurements. For the present study, we analyzed TL measurements for total lymphocytes. More detailed description of flow FISH methods used in this study can be found elsewhere [42].

DNA for qPCR assays was extracted with the QIAamp Maxi Kit procedure (Qiagen, Inc., Valencia, CA). We used *Telo_RP* and *Telo_FP* primers (for the telomeric PCR) and *36B4_FP* and *36B4_RP* primers for the single-copy gene (36B4). Raw T/S ratio was standardized by dividing the raw ratio with the average T/S ratio of internal quality control (QC) calibrator samples. All samples were measured in triplicate and averages were used in the final calculations. More detailed description of the qPCR method used in this study can be found elsewhere [42]. qPCR analysis was completed in stages; to ensure comparability between TOAA sub-cohorts, we standardized TL based on the TL distribution within the sub-cohorts using z-scores $\left(Z=\frac{x-\mu }{\sigma }\right)$ where X is the observed TL, *μ* is the mean TL, and *σ* is the TL standard deviation. Calculated z-score is interpreted as the number of standard deviations from the mean.

### Prediction of telomere length using DNA methylation array data

For DNA methylation profiling, we used TOAA blood extracted DNA and the Illumina Infinium Methylation EPIC Bead™ array which covers more than 850,000 methylation sites across the genome. We excluded samples where >4% of probes failed detection (n=2). Functional normalization was used to account for potential batch effects using the “minfi” R package. Forty-eight blinded duplicate samples included to assess within and across plate differences had a high concordance rate (Pearson’s r ≥ 0.98). The Horvath DNAmTL was used for TL estimation as previously published [22]. In the original study, machine learning techniques were used to identify CpG sites (cytosine-phosphate-guanine dinucleotides) predictive of TRF-measured leukocyte TL; this resulted in the selection of 140 specific CpG sites using data from a diverse sample of 2,256 individuals. The use of linear regression models allowed for the transformation of DNA methylation levels to express TL predictions in kb.

### Statistical analysis

We used Pearson’s correlation coefficient to evaluate the strength of linear association between DNAmTL and other TL measurement methods (flow FISH, and qPCR). A multivariable linear regression model was used to assess the association between DNAmTL and participant characteristics (age, sex, race, and CMV serostatus). All tests were two-sided with statistical significance defined as p < 0.05.

Bland Altman analysis was used to assess agreement between TL measurement methods by studying the mean difference between the two methods and constructing limits of agreement (defined as area within two standard deviations of the mean difference). The Y-axis shows the measurement difference, and the X-axis represents the average of the two measures. Bias is estimated as the mean difference between TL measurements in each comparison, with the zero line representing perfect agreement [43]. We used a classifier matrix (also known as confusion matrix) to assess the ability of qPCR and DNAmTL to accurately classify categories of TL in comparison with flow FISH using the R package (caret). Performance metrics included accuracy, sensitivity, specificity, and negative and positive predictive values. Data analysis and visualization was performed using SAS® statistical software, version 9.4 and RStudio, version 1.3.959.

### Data availability

Data from this study are available and can be shared after fulfilling data sharing requirements; all relevant data and methods are reported in the article.
